# A case report of early diagnosis of asymptomatic hairy cell leukemia using flow cytometry

**DOI:** 10.3389/fimmu.2023.1207443

**Published:** 2023-05-22

**Authors:** James Tadros, Amanda Davis, Oreoluwa Awoleye, Evros Vassiliou

**Affiliations:** ^1^ Colfax Oncology, Paterson, NJ, United States; ^2^ Department of Biological Sciences, Kean University, Union, NJ, United States

**Keywords:** hairy cell leukemia, flow-cytometry, immunophenotyping, oncology, B-cell

## Abstract

Hairy Cell Leukemia is an infrequent leukemia that can be recognized both microscopically and flow cytometrically once the patient develops symptoms. We present a case where early diagnosis was achieved using flow cytometry long before the patient became symptomatic. This was achieved by focusing on a small percentage (0.9%) of total leukocytes that exhibited a higher side scatter and brighter CD19/CD20 than the remaining lymphocytes. A bone marrow aspirate three weeks later confirmed the presence of malignant B-cells. Shortly after, the patient presented splenomegaly and complained of fatigue.

## 1 Introduction

Hairy cell leukemia (HCL) is a rare mature B-Cell neoplasm, comprising 2% of Lymphoid Leukemias. It is commonly found in middle-aged and elderly men (5:1 ratio) (1–3). Patients usually present with cytopenia and an enlarged spleen. The characteristic cells are mononuclear with abundant cytoplasm, with projections extending over the cell surface with round or oval nuclei, observed in the peripheral blood, infiltrating the bone marrow and in the pulp of the spleen (4–6). Flow cytometric immunophenotyping characteristically demonstrates a monoclonal population of mature B cells with expression of CD25, CD11c and CD103. These markers characteristically distinguish HCL from other CD5 negative B-cell lymphoproliferative disorders like splenic, villous and marginal zone lymphoma (7, 8). Variant forms and atypical HCL may express different phenotype, like absent CD103 or CD25 or variable intensity of CD11c (9, 10). Some rare cases were found to have CD5 positive (11). Cytogenetic studies can also be helpful in identifying those cases of HCL with aberrant cell markers since recently BRAF V600E mutation has been reported to be associated with the atypical form (12–14). Gene profiling has demonstrated clearly that HCL displays a homogenous pattern of expression which is distinct from that of other B-cell non-Hodgkin lymphomas (13).

## 2 Case history

The patient is a 41 year old male with low platelets for Hematology consult referred for thrombocytopenia. CBC report revealed WBC 4.2 K/μL, RBC 5.12 M/μL, Hgb 15.4 g/dL, Hct 45.7%, MCV 89 fl, MCH 30.1 pg, MCHC 33.7 g/dL, RDW 13.6%, platelets 120K/μL with differential count of granulocytes 59.4%, Lymphocytes 36.8%, monocytes 3.8%.

## 3 Flowcytometric studies

Flow cytometry was performed for CD5, CD10, CD19, CD20, CD22, CD25, HLA-DR, FMC7, CD45, CD103, Kappa and Lambda. All antibodies were obtained from BD Bioscience (San Jose, CA). Files were acquired with a BD FACScanto II instrument.

## 4 Discussion

**Figure 1:**
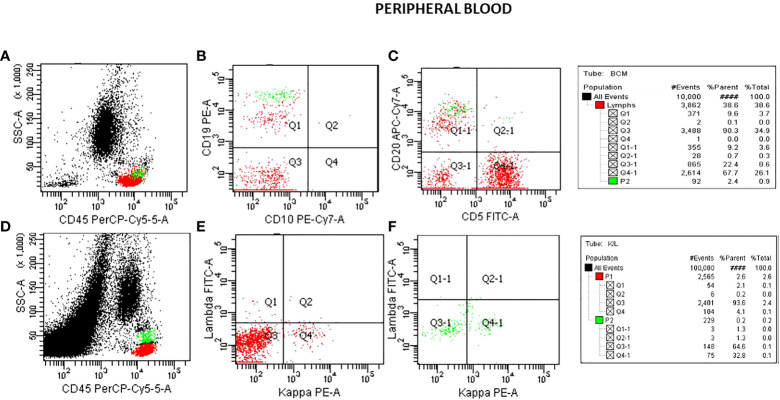
Flow cytometry analysis on peripheral blood specimen. Plots for CD45/SSC **(A)**, CD10/CD19 **(B)**, CD5/CD20 **(C)** showing a B-cell population about 10% of the lymphocytes with a small population (P2 green) with brighter CD19 and CD20 than the rest of the B-cell population. A plot of CD45/SSC **(D)** with a collection of 1,000,000 cells shows the magnified CD19 population (P2). This shows K/L in the small population as 32.8:1.3 monoclonal **(F)**. K/L in the total B-cells (P1) shows as 4.1:2.1 polyclonal **(E)**.

**Figure 2:**
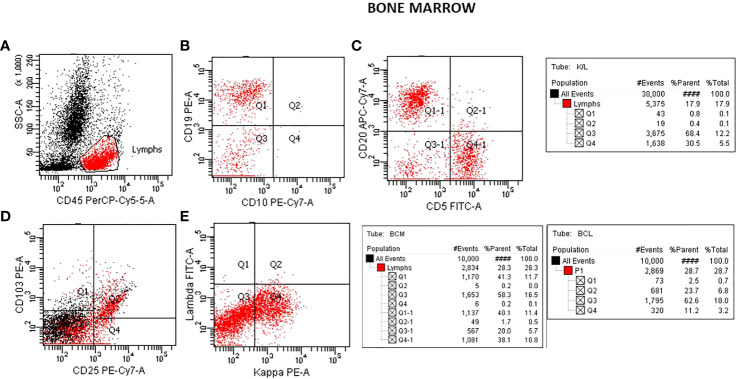
Flow cytometry analysis on bone marrow aspirate specimen. Plots for CD45/SSC **(A)**, CD10/CD19 **(B)**, CD5/CD20 **(C)** showing approximately 40% of the lymphocytes with CD19, CD20 (B-cells). A plot showing positive CD25/CD103 **(D)** seen in Hairy Cell Leukemia and a plot showing K/L with mostly Kappa 30.5:0.8 monoclonal **(E)**.

**Figure 3:**
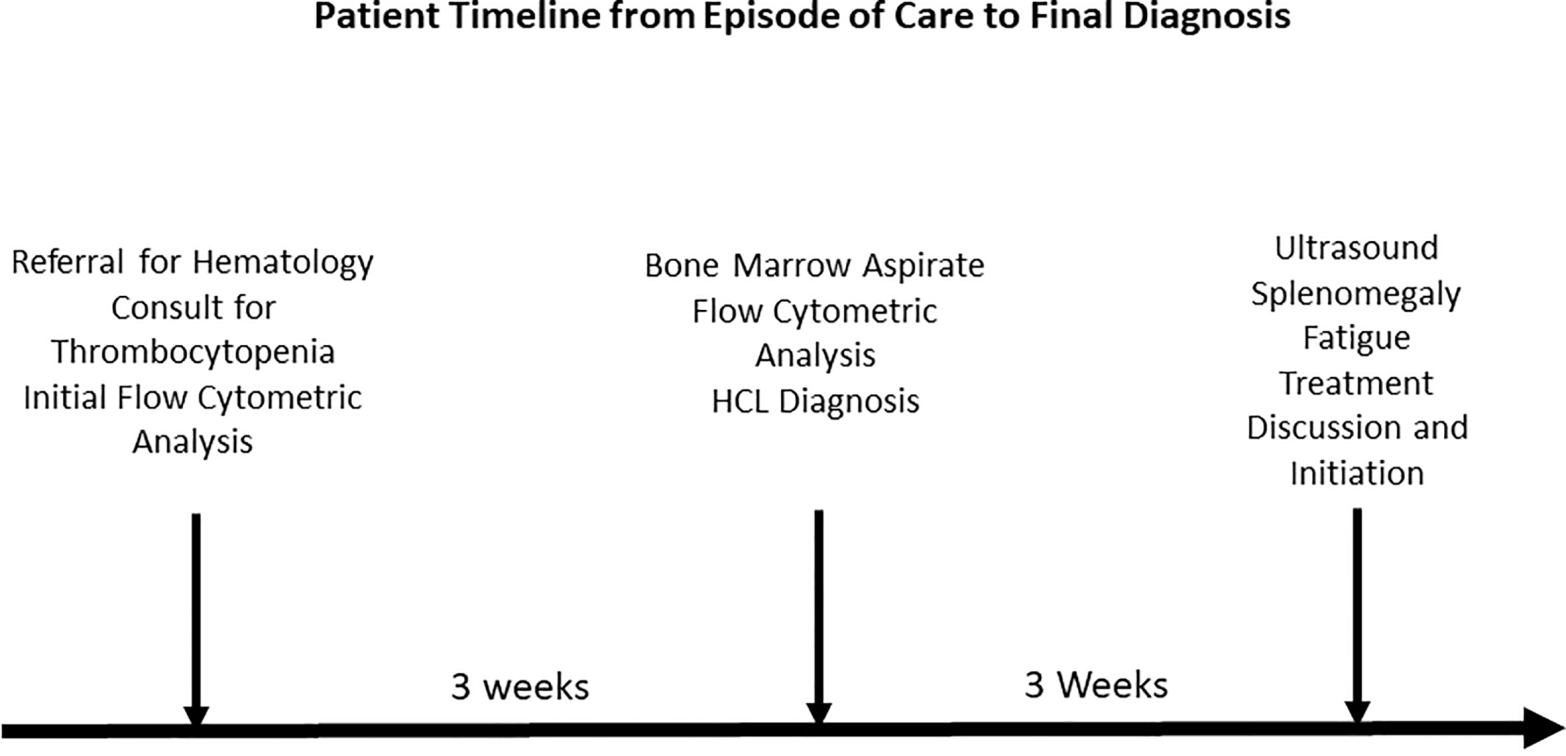
Patient timeline from episode of care to final diagnosis.

Flow cytometric analysis was performed by initially plotting CD45 vs SSC, gating on the lymphocyte population, and then gating on the brighter than normal CD19 and CD20 sub-population. Lastly, the gated population was evaluated for kappa/lambda monoclonality. The peripheral blood analysis showed a lymphocyte population of about 38.6% with no apparent abnormal antigen expression (**Figure 1**). The B-cells were approximately 10% of the lymphocytes and appeared polyclonal. There was however a small population (about 2.4% of all the lymphocytes) with a slightly higher side scatter that showed brighter CD19, and CD20. By collecting about 1,000,000 cells in all events, it showed monoclonal kappa light chain, while the remainder of the B cells were polyclonal. Further study for this small population was recommended with emphasis on Bone Marrow biopsy and comprehensive pathologic and cytogenetic studies. A bone marrow aspirate was submitted after about 3 weeks of the initial study on the peripheral blood and now revealed a lymphocyte population of 34% of the total with B-cells approximately 41% of the lymphocytes and the entire population was positive for CD19, CD20, CD22, HLA-DR, FMC7, and kappa monoclonal (**Figure 2**). The population also appeared to be partially positive for CD25 and CD103. Review of the bone marrow morphology demonstrated normocellular marrow with B-cell infiltrate representing 30-40% of the marrow cellularity that were predominantly medium sized mature forms with oval-shaped nuclei and appreciable cytoplasm with cytoplasmic projections. The B cells were positive for CD20, Annexin A1, DBA44, BCL1, BCL2, TRAP and BRAF V600E, but negative for CD5, CD10, LEF1 or BCL6. Overall the findings were consistent with Hairy Cell Leukemia. On follow-up, the patient showed splenomegaly by ultrasound and started to complain of fatigue (**Figure 3**).

## 5 Conclusion

By investigating thoroughly the small percentage (about 1% of the total cells), we were able to diagnose HCL (Hairy Cell leukemia) in its early stages. The patient was informed and treatment outcome was favorable. The higher than usual side scattering of the lymphocytes would have gone unnoticed, if the typical 50,000 events were analyzed. Adjusting to 1,000,000 events allowed for more scrutiny of the neoplastic subpopulation. This case report is an excellent example that highlights the superiority of flow cytometric analysis in comparison to other techniques. It is an important diagnostic tool that can influence therapeutic decisions and disease progression in a variety of clinical settings (15).

## Data availability statement

The original contributions presented in the study are included in the article/supplementary material. Further inquiries can be directed to the corresponding author.

## Ethics statement

Ethical review and approval was not required for the study on human participants in accordance with the local legislation and institutional requirements. The patients/participants provided their written informed consent to participate in this study.

## Author contributions

Conceptualization, JT; writing—original draft preparation, JT and EV; writing—review and editing, JT, EV, OA, and AD. All authors contributed to the article and approved the submitted version.
